# Notes and News

**Published:** 1888-05-05

**Authors:** 


### NOTES AND NEWS.

Collected and Communicated.

At the Shadwell Hospital out of 1,018 in-patients 456 were under two years of age, and 20 were under one month.

A "Church Parade" by the local friendly societies is to be held on Sunday, May 13th, for the benefit of the Grantham Hospital.

Last week the Marchioness of Waterford opened a bazaar at the Athenæum, Camden Road, in aid of the funds of the North-West London Hospital.

Lord Justice Bowen presided on Monday at the annual festival dinner of the King's College Hospital. Donations to the amount of £2,000 were announced.

Colonel and Mrs. Seely have offered their house, Sherwood Lodge, with the use of horses and carriages, dairy and garden produce, for the reception of twenty convalescents, with nurses and servants, from the Nottingham Children's Hospital.

Mr. Thomas W. Mason has written to the *Suffolk Chronicle* asking for toys, or subscriptions towards purchasing them, for the children's wing of the East Suffolk Hospital. It seems that, with the exception of a rocking-horse and a boat, there is not a toy of any sort in the ward—a state of things fortunately very uncommon.

His Royal Highness the Duke of Cambridge will preside at the annual festival dinner of the Middlesex Hospital, which will take place at the Hôtel Métropole on Tuesday, May 15th. Many noblemen and gentlemen have consented to act as stewards, and it is hoped that a highly successful festival will be the result.

The thirty-sixth anniversary dinner of the Hospital for Sick Children was held on the 25th ult. at Willis's Rooms. The Bishop of Peterborough, who presided, remarked in his speech that the assured income of the hospital was only £417, as against an expenditure of nearly £10,000, but he added that perhaps the great secret of the prosperity of such an institution was to be constantly on the verge of bankruptcy.

Professor Bergmann, it is announced, has written to Prince Radolin, tendering his resignation as a member of the Emperor Frederick's medical staff. The behaviour of several heretofore illustrious medical men in connexion with the illness of the Emperor contrasts painfully with his own dignified and heroic patience. It is a disgrace and a scandal to medicine throughout the world, and makes English practitioners in the remotest villages blush for shame.

An attempt was made on Sunday to blow up the Small-pox Hospital, recently erected at Troopers' Hill, near Bristol, with gunpowder. There has for some time been a strongly antagonistic feeling against the erection of the hospital in the neighbourhood, and this culminated on Sunday in the forcible measure of attempted destruction. Fortunately the would-be Guy Fawkes was a clumsy manipulator, and very little harm was done.

It has been resolved that scarlet fever cases shall no longer be treated at the Birmingham Children's Hospital, as the demand for accommodation for infectious cases has greatly diminished since the establishment of the Borough Hospital, and the managing committee feel also that it is better to prevent the risk of scarlet fever spreading in the hospital. Typhoid and diphtheria cases will, however, continue to be admitted.

Mr. A. W. Webster read a paper to the managers and friends of the Hospital Saturday Movement at the Memorial Hall, Farringdon Street, on Monday evening. Mr. H. Hamilton-Hoare presided. Mr. Webster pointed out the great results which had flowed from the adoption of the penny a-week system in Sunderland, and advised its being tried in London. The London managers did not encourage the use of the system in London on account of the difficulties of collection. Must we assume that those difficulties are absolutely insuperable?

A concert was given on Saturday at Grosvenor House by permission of the Duke of Westminster, in aid of the funds of the South London District Nursing Association, for nursing the poor in their own homes, in connexion with the Metropolitan and National Nursing Association. The performers were the Royal Amateur Orchestral Society, of which the Duke of Edinburgh is president, with Mr. George Mount as conductor and Mr. J. F. Bambridge as accompanist, Lady Trelawny, Lady Ramsay, Lady Sandhurst, Lady Randolph Churchill, Miss Emma Allitsen, Mrs. Ronalds, Mr. Arthur Lionel Scott, Mr. John V. Croft, Mr. Ernest Ford, and Mr. John Thomas.

The Mayor of Bristol presided at the annual meeting of the Bristol Children's Hospital on the 24th, and Mr. Mark Whitwell, the president and treasurer, read the report. The institution is in a progressive condition. The work done was more, the income larger, and the expenditure also considerably in excess of former years. The financial year commenced with a credit balance of £18 18*s*. 4*d*., and closed with a debit of £93 6*s*. 9*d*. That is to be regretted, but not too much mourned over. The credit should be restored in the current year. Among the items of income there is a sum of 10*s*. 2*d*. arising from a "sale of kittens." Mr. John Lockhart Livingstone, M.D., has been appointed house-surgeon, and Mr. Lawford Jones secretary and collector.

The governors of St. Thomas's Hospital, the Corporation representatives of course included, will doubtless be only too glad if the prophecy of an enthusiastic Orpingtonian should prove quickly correct. St. Thomas's Hospital possesses a fine estate close to Orpington Station, and at some period it is likely to become, in the language of Tokenhouse Yard, ripe for building. The enthusiastic Orpingtonian, whose belief is that Chiselhurst has "had its day"—what will Sir John Bennett say to this heresy?—thinks that Orpington, being the next place southward from London, is bound shortly to move, and become part and parcel of the metropolitan outskirts. When that time comes the governors of St. Thomas's can look for a right royal revenue from ground rents and so forth. Mr. Ruskin is not the one to hold that a consummation devoutly to be wished, albeit he is assisting to make Orpington known to fame by using it as the centre, instead of Paternoster Row, for the distribution of his remarkable works.

THE following vacancies are announced this week, the amount of salary in each case being given after the name of the institution :—

**Resident Medical Officers.**

Ancoats Hospital. Junior Visiting Surgeon —£60.
Birmingham General Dispensary. Resident Surgeon. –£150.
Bury Dispensary Hospital. Junior House Surgeon.—£60.
Croydon General Hospital. House Surgeon.—£100.
Hertford British Hospital, Paris. House Surgeon.
Hull Royal Infirmary. Junior Assistant House Surgeon.—£50.
Hospital for the Paralysed and Epileptic, Queen Square. House Physician.—£50.

**Non-resident Medical Officers.**

Brighton Medical Officer of Health.—£500.
Callan Union. Medical Officer.—£150.
Durham County Hospital. Honorary Surgeon.
London Throat Hospital, Great Portland Street. Surgeon.

---

A VERY successful bazaar has been held at Hereford for the benefit of the Victoria Eye and Ear Hospital there.

---

MR. JAMES STEPHEN BLYTH, Secretary of the Royal Free Hospital, which post he had held for nineteen years, died at Sydenham on the 22nd of April, and was buried at Norwood on Friday, the 27th ult. Mr. Blyth was popular with the committee and medical staff, both being represented at the funeral. Dr. Steele, of Guy's Hospital, attended as the representative of the Hospitals Club, Mr. Alexander Marsden, F.R.C.S., the son of the founder of the Royal Free Hospital, and Mr. William Rose, F.R.C.S., the eminent surgeon, being also present.

---

THE women of India and their friends in this country have reason to congratulate each other on the success which has attended Lady Reay's bazaar and fancy fair. The fair was held in support of Lady Dufferin's movement for supplying skilled medical aid to Indian women. Many people are beginning to know of Lady Dufferin's movement, and it would be well if many more would take a little pains to inform themselves. This country owes vast debts to India, and India owes something to us. But the really wholesome-hearted man or woman does not ask what obligations he is bound to fulfil in order to make his conscience easy, but what powers and resources are at his disposal which may be used for the service of his fellows. All who have any direct or relative connection with India, and particularly women, should stir themselves up to a carefully-intelligent understanding of Lady Dufferin's movement. It appeals both to the imagination, the reason, and the sympathy of gentle and generous souls. The poor and the rich Indian women are alike in a grievous plight when dangerously ill. Skilled medical aid, as we understand it, they have none. But what does skilled medical aid mean ? Nothing less than this—a relief of suffering and a saving of life which are incalculable. European medicine in these times is not charlatanism and luck ; it is science, reason, practical skill, the adaptation of tried and proved methods to the production of definite results. This kind of aid, which British hospitals place at the disposal of the very poorest in this country, the wealthiest and the worthiest of Indian women cannot obtain. Lady Reay's fancy fair has yielded a net profit of 43,000 rupees—a splendid result. Towards this amount the Duchess of Connaught's stall contributed 5,000 rupees ; Lady Reay's own stall the noble total of over 8,000 ; and Lady Morland's Café Chantant nearly 4,000. The whole receipts amounted to 49,000 rupees, and the expenses were only 6,000, or about 16 per cent. ; a very small sum indeed compared with the usual expenditure in such cases. The cause which Lady Dufferin has in hand, and her thoroughly capable and successful management of it, are worthy of the sincerest admiration.

THE *Westminster Review* for May contains an article on "Habitual Drunkenness," by an Habitual Drunkard. According to the writer, total abstinence is the only possible cure.

---

A CORRESPONDENT of the *Sunderland Herald*, who signs himself "Ghost," and claims to be the spirit of a martyred Christian of Apostolic times, gives the following earnest and wise advice : "Do not preach the glories of heaven to those who are dying for want of the necessities of earth. Do not preach to the undisciplined soul perishing for bread that heavenly bread in abundance is waiting for those who are lucky enough to get it. Lastly, remember that a man who fares sumptuously every day is not a Christian so long as he forgets to minister to the wants of the hungry, and thirsty, and naked." As the letter is headed "Infirmary Collections," it may be feared that the rich men of Sunderland are not so considerate of the wants of the suffering as their workmen.

---

MRS. PHILLIPS, matron of the Kidderminster Infirmary, has presented a stained window to the institution, as a memento of her long connexion with it, she having come to the infirmary in 1873. The subject chosen is a most appropriate one—"The Good Samaritan"—and the work has been most efficiently carried out, the harmony of colour being very effective. The central figure is the Good Samaritan in the act of pouring oil into and binding up the wounds of the unfortunate man who has fallen among thieves ; and in the background has been introduced a representation of the Eastern Mosque, with a view of Eastern customs. At the foot of the window are the words : "He bound up his wounds ;" and underneath is a brass plate bearing the following inscription : "This window is presented by Mrs. Phillips, in commemoration of her fifteen years' residence as Matron. Easter, 1888." The work has been carried out by Messrs. Gibbs and Howard, of London, who are also engaged on a larger window for the main staircase.

---

"Two thoroughly equipped horse-ambulances" constituted the noble Jubilee present given by the working-men of Leeds to the Royal Infirmary of that town last year. "The large increase of income," says the Annual Report, "derived from workpeople's contributions is a matter for much satisfaction." The total amount raised by the workpeople during 1887 was £3,692, as compared with £1,884 in 1886—an increase of £1,808. How did that increase come about ? Not because trade was better ; not because the town was larger ; but because the organisation by which the money was collected was made worthy of the occasion. The Leeds workpeople have probably been as able to give nearly four thousand a-year any time the last twenty years as they are now. They have not perhaps given more than a quarter of that amount. There may have been a loss to the infirmary of not less than £50,000 during the twenty years, and for what reason ? Because there has never been sufficient troubl taken to put the claims of the institution before the workers clearly and convincingly. We do not say these things to reproach Leeds and the infirmary managers : far from it -they deserve all credit in that they have at last opened out and worked the gold mine which has so long been neglected, to the injury both of the workpeople and the hospital. We give them prominence in order that other hospital managers and officials in other towns, and particularly in London, may see what has been done in Leeds, and set themselves to produce like results by the adoption of like methods. There has been a steady falling-off in Leeds for some years in the Hospital Sunday collections ; but that has been more than compensated by the enormous growth of the workpeople's fund. That working-men should help themselves is in every respect desirable and creditable ; and we hope that the way so clearly shown by Leeds will soon be traversed by all the hospitals and working-men in Great Britain.

